# High PER1 expression is associated with *STK11* mutation and clinical biomarkers of immunotherapy resistance in lung adenocarcinoma

**DOI:** 10.1007/s00432-025-06269-9

**Published:** 2025-07-26

**Authors:** Rebecca E. Parker, Leon McSwain, Wei Zhou, Adam I. Marcus, Haian Fu, Suresh S. Ramalingam, Shirley Zhang, Melissa Gilbert-Ross

**Affiliations:** 1 Cancer Biology Graduate Program, Graduate Division of Biological and Biomedical Sciences, Laney Graduate School, Emory University, Atlanta, GA USA; 2 Department Hematology and Medical Oncology, Emory University School of Medicine, Atlanta, GA USA; 3 Winship Cancer Institute of Emory University, Atlanta, GA USA; 4 Department of Pediatrics, Emory University School of Medicine, Atlanta, GA USA; 5 Department of Pharmacology and Chemical Biology, Emory University School of Medicine, Atlanta, GA USA; 6 Department of Cell Biology, Emory University School of Medicine, Atlanta, GA USA

**Keywords:** LKB1, STK11, PD-L1, PER1, Lung adenocarcinoma, Immunotherapy, CD274, Invasion

## Abstract

**Aim:**

This study characterizes the functional effects and clinical characteristics of high *PER1* mRNA and high PER1 protein expression in treatment resistant lung adenocarcinoma.

**Methods:**

HBEC3-KT cells were modified by *STK11* CRISPR knockout and A549 cells by LKB1 addback using stable transfection. RNA sequencing and western blot were used to profile gene and protein expression. Pooled siRNA knockdown of PER1 was used to assess impacts on cell proliferation and 3D invasion. Human lung adenocarcinoma data were analyzed using cBioPortal.

**Results:**

*PER1* mRNA and protein are upregulated in *STK11*-mutant lung adenocarcinoma tumors and *STK11*-knockout human bronchial epithelial cells (HBEC3-KT). Addback of wildtype LKB1 in A549 lung cancer cells is sufficient to decrease PER1 protein levels. Knockdown of PER1 decreased cell growth, proliferation, and 3D invasion in LKB1-deficient cell models. High *PER1* expression in lung cancer patients correlates with LKB1 mutation status, decreased expression of the gene that encodes PD-L1, and altered hypoxia and immune and stromal ESTIMATE scores.

**Conclusion:**

PER1 has oncogenic activity in LKB1-mutant lung cancer cells and high *PER1* expression in lung adenocarcinoma patients may represent an independent biomarker of resistance to immunotherapy.

**Supplementary Information:**

The online version contains supplementary material available at 10.1007/s00432-025-06269-9.

## Introduction

Lung cancer treatment and prognosis is highly dependent on molecular subtype, and *KRAS* mutations are the most common genetic driver. Within *KRAS*-mutant lung cancer, three subtypes are defined by co-mutation of *TP53*, *STK11*, or other genes and these subtypes have critical implications for prognosis and treatment options (Skoulidis et al. 2015, 2018; Skoulidis and Heymach 2019). *STK11* encodes the LKB1 serine/threonine kinase that activates AMPK-family kinases to regulate metabolism, cell polarity, cell migration, and the DNA damage response (Hawley et al. 2003; Faubert et al. 2014; Shackelford and Shaw 2009; Wang et al. 2016; Gupta et al. 2015; Wilkinson et al. 2017; Konen et al. 2016; Boudeau et al. 2003). LKB1 activity is frequently compromised by somatic mutation and epigenetic regulation in lung adenocarcinoma, and *KRAS*/*STK11* co-mutation results in worse prognosis than other *KRAS*-mutant subtypes due to a greater likelihood of metastasis and resistance to standard-of-care treatments, including immune checkpoint inhibitors (Skoulidis et al. 2018; Calles et al. 2015; Rosellini et al. 2022). Because of its myriad roles, loss of LKB1 drastically changes cell and tumor biology, necessitating subtype-specific discovery of novel targets and development of new therapeutics for this aggressive disease.

We previously reported that *KRAS*-mutant/LKB1-deficient lung adenocarcinoma patients show altered AMPK-regulated gene circuits related to biological rhythms (Rackley et al. 2021), which are controlled by the circadian clock. The core circadian clock machinery is comprised of the positive transcriptional activators CLOCK and BMAL1 that heterodimerize to drive rhythmic transcription of *PER* and *CRY* genes, as well as other clock-controlled genes. In turn, the PER/CRY protein complexes translocate into the nucleus to inhibit CLOCK/BMAL1 (Partch et al. 2014). An additional loop involving RORs and REVERBs controls the expression of *BMAL1*, and the output of these intersecting loops is the rhythmic expression of clock-controlled genes over a 24-h period. Importantly, the circadian clock controls rhythmic expression of gene products that regulate cell cycle, DNA repair, metabolism, cell migration and cell invasion (Shafi and Knudsen 2019), thus having critical implications for multiple treatment modalities (Sulli et al. 2019; Masri and Sassone-Corsi 2018).

Generally, when data is analyzed without consideration of specific driver mutations or molecular subtype, PERs have been shown to possess tumor suppressor activity in a variety of cancers, with low *PER1* expression associated with poorer prognosis in lung cancer patients taken as whole (Zhang et al. 2020; Cadenas et al. 2014; Deng et al. 2020; Chen et al. 2021; Gery et al. 2007). In a murine study of *Kras*/*Trp53*-mutant lung cancer, deletion of *Per2* within tumors accelerated tumor progression, indicating a tumor suppressive role for PERs in this subtype (Papagiannakopoulos et al. 2016). Disruption of the central circadian pacemaker through jet lag also accelerated tumor growth in this *Kras*/*Trp53*-mutant model, suggesting that normal rhythmic function of the central circadian clock in the brain plays a similar role during tumor progression. However, when considering *STK11*-mutation status in studies that did not originally focus on this important variable, the role of PERs appears to be oncogenic. For example, in an *STK11*-mutant model, PER1 has been shown to interact with and destabilize p53, functionally reducing sensitivity of lung cancer cells to DNA-damaging therapeutics (Bellet et al. 2021). Thus, PER1 may have unique subtype-specific roles in lung cancer with critical implications for treatment efficacy.

The objective of this study was to investigate *PER1* mRNA expression and PER1 protein expression in LKB1-deficient cell models and lung cancer patient samples and to assess the functional impact and clinical significance of high *PER1* and PER1 expression in LKB1-deficient lung cancer.

## Materials and methods

### Cell lines and cell culture conditions

The HBEC3-KT cell line (RRID:CVCL_X491) was a gift from Dr. John Minna (The University of Texas Southwestern Medical Center, Dallas, TX) and was cultured in Keratinocyte serum-free medium (K-SFM; Gibco, 17,005,042) with supplied recombinant epidermal growth factor and bovine pituitary extract added. The KRAS and shLKB1 HBEC3-KT cell lines were produced as described previously (Koo et al. 2024). The LKB1-wildtype and LKB1-knockout HBEC3-KT clonal populations were produced by Synthego, in consultation with the Emory Integrated Genomics Core facility, using their antibiotic-free CRISPR/SpCas9 single guide knockout method. The guide RNA sequence used was CGAUGAGCUUGGCCCGCUUG.

The A549 cell lines (RRID:CVCL_0023) were a gift from Dr. Wei Zhou (Winship Cancer Institute of Emory University, Atlanta, GA) and produced as previously described (Liu et al. 2015). The H1299 cell lines (RRID:CVCL_0060) were a gift of Dr. Adam Marcus (Winship Cancer Institute of Emory University, Atlanta, GA) and produced as previously described (Konen et al. 2017). The JK-43-M cell line was produced in our lab as previously described (Koo et al. 2024). The A549, H1299, and JK-43-M cell lines were maintained in RPMI 1640 medium with L-glutamine (Corning, 10–040-CV) supplemented with 10% fetal bovine serum (FBS; Corning, 35–011-CV) and 1% PenStrep-glutamine (Gibco, 10,378,016). For experiments, cells were cultured in RPMI-1640 with 10% FBS and without antibiotics.

All cell lines were tested and confirmed negative for mycoplasma (Universal Mycoplasma Detection Kit, ATCC, 30-1012 K) and used for experiments within three months of thawing.

### Glucose starvation assay of LKB1 activity

Though other kinases, such as CAMKK2, can phosphorylate AMPK at the T172 position, only LKB1 phosphorylates AMPK at T172 in response to glucose starvation; therefore, a glucose starvation assay can be used to test whether LKB1 is functional in a cell line. We cultured cells in their typical complete culture media and plated them onto 10-cm dishes and allowed them to grow until approximately 90% confluence. Complete medium was aspirated and replaced with either RPMI with 2 mg/L glucose (Corning, 10–040-CV) or RPMI without glucose (Corning, 10–043-CV); of note, these media were not supplemented with FBS. Samples were collected after 4 h of incubation in a cell culture incubator (21% O_2_, 5% CO_2_, 37 °C) and processed for western blot as described below.

### siRNA knockdown of PER1

Dharmacon ON-TARGETplus siRNA SMARTPool reagents were used. The siPER1 SMARTPool (Horizon Discovery, L-011350–00-0005) targeted the following sequences: CCAAUAAGGCGGAGAGUGU, CCAGUGACCUGCUCGAACU, GGCCGAAUCGUCUACAUUU, and CAACGGGCAUGAGUCUAGA. As a negative control, the ON-TARGETplus non-targeting control pool siRNA (siNTC) was used (Horizon Discovery, D-001810–10-05) and targeted the following sequences: UGGUUUACAUGUCGACUAA, UGGUUUACAUGUUGUGUGA, UGGUUUACAUGUUUUCUGA, and UGGUUUACAUGUUUUCCUA. The transfection reagent used was DharmaFECT 1 (Horizon Discovery, T-2001–02). Transfections for all cell lines were conducted using the reagent concentrations provided in the Dharmacon protocol for A549 cells.

### Invasion assays

HBEC3-KT 3D invasion assays were performed as previously described (Koo et al. 2024). H1299 3D invasion assays and image analysis were performed as previously described (Konen et al. 2017), with cells treated with siRNA 24 h prior to spheroid formation via centrifugation of cells in ultra-low attachment 96-well plates (Corning, 7007).

### Western blot analysis and antibodies

To prepare whole cell lysates, cells were washed with ice-cold phosphate-buffered saline (PBS), then scraped into 1 ml of fresh ice-cold PBS and transferred into a 1.5 ml tube. Cells were pelleted by centrifugation (2000 × g for 5 min at 4 °C) and the cell pellet was then resuspended in high-salt NP-40 lysis buffer (Alfa Aesar, J61428) supplemented with a protease and phosphatase inhibitor cocktail (Cell Signaling, 5872), incubated on ice for 10 min, then briefly sonicated to shear DNA. To prepare subcellular fractionation lysates, the REAP method (Suzuki et al. 2010) was used with 100 μl volume in each fraction, sonication of all samples, and addition of protease and phosphatase inhibitor cocktail (Cell Signaling, 5872) to finished fractions. Protein concentration was measured using Bradford assay (Bio-Rad, 5,000,205) and samples with equal protein concentrations were prepared using homemade 5X Laemmli buffer supplemented with 10% beta-mercaptoethanol and then boiled for 5 min. After electrophoresis, gels were transferred overnight onto PVDF membrane (Bio-Rad, 1,620,177). Membranes were blocked in TBS-T containing 5% non-fat milk powder (Cell Signaling, 9999; Apex, 20–241) and 5% BSA (Cell Signaling, 9998).

The following primary antibodies were diluted in 5% BSA in tris-buffered saline (Cell Signaling, 12498S) supplemented with 0.01% Tween-20 (TBS-T; ChemCruz, sc-29113B) and used at the indicated dilutions: PER1 (1:1000, Boster Bio, A00876, RRID:AB_3086701), LKB1 (1:1000, Cell Signaling, #3050, RRID:AB_823559), JAG1 (1:1000, Cell Signaling, #70,109, RRID:AB_2799774), pAMPK-T172 (1:1000, Cell Signaling, #2535, RRID:AB_331250), AMPK (1:1000, Cell Signaling, #2532, RRID:AB_330331), Actin (1:500, DSHB, JLA20, RRID:AB_528068; 1:1000, Sigma-Aldrich, A5441, RRID:AB_476744), Lamin A/C (1:1000, DSHB, MANLAC3(4C10), RRID:AB_2618205). The following HRP-conjugated secondary antibodies were diluted in 5% non-fat milk in TBS-T at a 1:3000 dilution: anti-Rabbit (Boster Bio, BA1054, RRID:AB_2734136) and anti-Mouse (Fisher Scientific, 62–652-0, RRID:AB_2533947). Blots were stripped after each successive primary antibody using OneMinute Plus Western Blot Stripping Buffer (GM Biosciences, GM6011). Blots were developed using SuperSignal West Pico PLUS chemiluminescent substrate (Thermo Scientific, 34,580) and imaged using a BioRad ChemiDoc.

### Cell proliferation assays

Cell proliferation assays were completed using two complementary methods: Cell Counting Kit-8 (CCK-8; Dojindo, CK04) and by manually counting trypsinized cells using a hemocytometer. For the CCK-8 method, after siRNA treatment HBEC3-KT cells were seeded in quadruplicate into multiple 96-well plates, one plate for each timepoint, at a density of 2000 cells per well and a volume of 100 μl per well. At each timepoint, 10 μl of CCK-8 reagent was added to each test well, incubated for 2 h, and then 450 nm absorbance was read using a microplate spectrophotometer (Biotek Epoch). For each condition, a control well without CCK-8 added was used to subtract background absorbance, leaving 3 wells as technical replicates.

For the cell counting method, cells were seeded, with HBEC3-KTs at a density of 2.5 × 10^4^ cells per well and A549s at a density of 2 × 10^4^ cells per well, in 24-well and 12-well plates, respectively. For the JK-43-M cells, 1 × 10^5^ cells were seeded into 6-well plates. At each timepoint, cells were trypsinized and resuspended in RPMI supplemented with 10% FBS to inactivate the trypsin, taking note of the total volume. Cells were counted using a hemocytometer to calculate total cell number per well.

### RNA-seq and gene expression analysis

RNA-seq experiments and analysis of H1299 parental, leader, and follower cells (Zoeller et al. 2019) and HBEC3-KT spheroids (Koo et al. 2024) were performed as previously described. For RNA-seq of the LKB1-WT and LKB1-KO HBEC3-KT cells, cells were plated in triplicate on 150 mm cell culture dishes and synced with a 30-min pulse of 100 nM dexamethasone. Samples were collected at 4 h increments from 24 to 48 h post-syncing. At each timepoint, the adhered cells were washed with ice-cold PBS, collected by scraping into ice cold PBS, pelleted, then flash frozen and kept at -80 °C until processing by the Emory Integrated Genomics Core. Total RNA was isolated using the miRNeasy Mini Kit (Qiagen, 217,004) then quantified and quality-controlled using the 2100 BioAnalyzer (Agilent). RNA-seq was performed by DLS Hudson Alpha at a sequencing depth of 50 million paired-end reads. For data processing, sequence adaptors were trimmed from fastq files using cutadapt (RRID:SCR_011841) and TrimGalore-0.6.10 (RRID:SCR_011847). Sequences were then aligned to the *Homo sapiens* reference genome release 111 using STAR genome aligner for paired end reads (RRID:SCR_004463). The resulting gene count data were normalized and differential expression analysis performed using DESeq2 (RRID:SCR_015687). QC inspection of the data resulted in one sample (KO_24_3) being removed from analysis. All timepoints were pooled for the analysis presented here.

### MCP-counter analysis of intratumoral immune cells

Full datasets of normalized gene expression for the TCGA-LUAD and CPTAC3 studies were downloaded from the Genomic Data Commons Data Portal (RRID:SCR_014514) for further analysis. The MCPcounter R package (Becht et al. 2016) was installed from GitHub using the devtools package and used with default gene signatures. After calculation of MCP-counter scores using R version 4.4.1, the data from each study were subset to only the samples present in the PER1/*PER1* expression quartiles, sorted by group, and the data were then statistically analyzed by two-way ANOVA followed by Šídák's multiple comparisons test in GraphPad Prism 10.4.1.

### Statistical analysis

Aside from statistical test results for clinical data calculated within cBioPortal, or others as indicated, all other statistical analyses were performed using GraphPad Prism 10.4.1 (627) (RRID:SCR_002798).

### Sex as a biological variable

Both male and female specimens from cBioPortal datasets were included in the analysis, but sexes were not analyzed separately or compared due to limited sample size and statistical power.

### Data availability statement

The RNA sequencing data analyzed in this study are available as follows: 3-D spheroid HBECs (NCBI Gene Expression Omnibus, GSE271368); H1299 parental, leader, and follower subpopulations (NCBI Sequence Read Archive, PRJNA542374); and synced time course of LKB1-WT and LKB1-KO cells **(**NCBI Sequence Read Archive, PRJNA1258988). Gene expression, protein abundance, mutation, and clinical data from human lung cancer specimens from the TCGA PanCancer Atlas, CPTAC 2020, and CPTAC GDC (July 2024) studies were accessed and downloaded through cBioPortal (RRID:SCR_014555, https://www.cbioportal.org/). Other data files will be provided upon request.

## Results

### LKB1 negatively regulates *PER1* transcript and protein abundance in partially transformed bronchial cell models and lung adenocarcinoma patient tumors

**Fig. 1:**
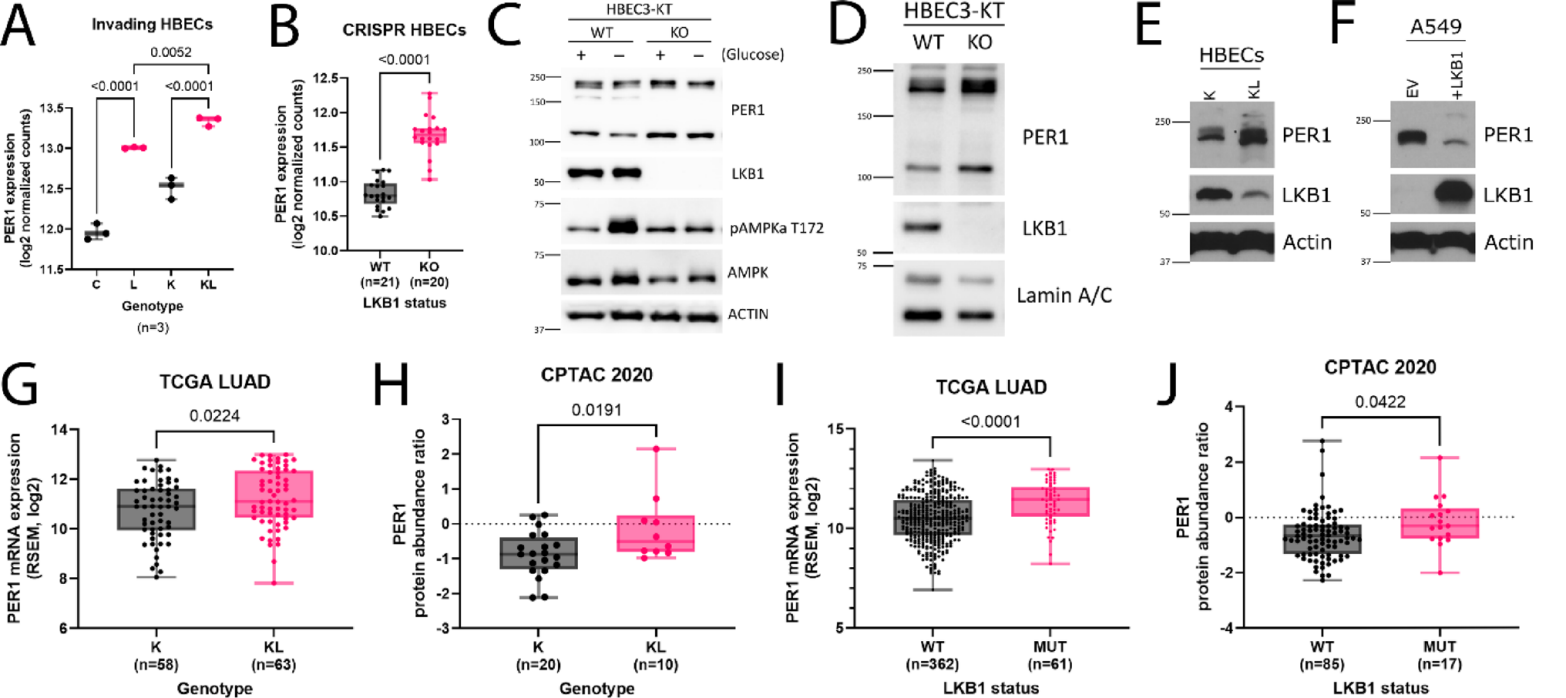
PER1 expression is increased with loss of LKB1. **A** *PER1* mRNA expression in control (C), sh*STK11* (L), KRAS^G12V^ (K), and KRAS^G12V^/sh*STK11* (KL) invading HBEC3-KT organoids; notated with adjusted p-values from ordinary one-way ANOVA followed by Šídák's multiple comparisons tests, C vs L p < 0.0001, K vs KL p < 0.0001, L vs KL p = 0.0052. **B** *PER1* mRNA expression in LKB1-wildtype (WT) and LKB1-knockout (KO) HBEC3-KT cells; two-tailed Student’s t-test, p < 0.0001. **C** Western blot of PER1, LKB1, pAMPK T172, AMPK, and Actin in LKB1-WT and LKB1-KO HBEC3-KT cells, with and without glucose. **D** Western blot of PER1, LKB1, and Lamin A/C in LKB1-WT and LKB1-KO HBEC3-KT cells. **E** Western blot of PER1, LKB1, and Actin in K and KL HBEC3-KT cells. **F** Western blot of PER1, LKB1, and Actin in empty-vector control A549 cells and with addback of wildtype LKB1. **G** *PER1* mRNA expression in *KRAS*-mutant (K) and *KRAS*/*STK11*-mutant (KL) tumors from the TCGA LUAD study; two-tailed Student’s t-test, p = 0.0224. H) PER1 protein abundance ratio in *KRAS*-mutant (K) and *KRAS*/*STK11*-mutant (KL) tumors from the CPTAC 2020 study; two-tailed Student’s t-test, p = 0.0191. I) *PER1* mRNA expression in LKB1-wildtype (WT) and LKB1-mutant (MUT) tumors from the TCGA LUAD study; two-tailed Student’s t-test, p < 0.0001. J) PER1 protein abundance ratio in LKB1-wildtype (WT) and LKB1-mutant (MUT) tumors from the CPTAC 2020 study; two-tailed Student’s t-test, p = 0.0422

To explore the relationship between LKB1 and core clock genes we analyzed a previously published RNAseq dataset from a panel of partially transformed and invasive human bronchial epithelial cell (HBEC) organoids (Koo et al. 2024) and found that shRNA knockdown of *STK11*, in both control and *KRAS*^G12D^-expressing cells, resulted in increased expression (p < 0.0001) of *PER1* transcript (Fig. 1A). In comparison, in this system knockdown of *TP53* decreased *PER1* expression (Figure S1A). To confirm these findings, we used CRISPR/Cas9 to knock out LKB1 in the same HBEC3-KT cell line, generating LKB1-wildtype (WT) and LKB1-knockout (KO) cells, and again found that loss of LKB1 resulted in an increase (p < 0.0001) in *PER1* mRNA expression (Fig. 1B). We confirmed that the LKB1-KO cells lacked LKB1 at the protein level and confirmed that the LKB1/AMPK signaling axis is non-functional using a glucose starvation assay (Fig. 1C). While the LKB1-WT HBEC3-KTs were able to phosphorylate AMPK at T172 in response to glucose starvation, LKB1-KO HBEC3-KT cells were not, indicating that the LKB1/AMPK signaling axis is inoperative in these cells. We also observed an increase in PER1 protein levels in these LKB1-KO HBEC3-KTs relative to LKB1-WT cells (Fig. 1D). Of note, PER1 is a highly regulated and post-translationally modified protein (Miyazaki et al. 2004; Narasimamurthy and Virshup 2021; Yang et al. 2012; Hirano et al. 2016) and in our western blot assays (as confirmed by siRNA knockdown; Figure S2) appears mainly in two bands of approximately 120 and 220 kDa. The 120 kDa band is consistent with the predicted molecular weight and the 220 kDa band has been reported to be a poly-ubiquitinated PER1 which is localized in the nucleus (Yang et al. 2012). Echoing our results in the LKB1-WT and LKB1-KO HBEC3-KT cells, in *KRAS*^G12V^-expressing partially transformed HBEC3-KTs, we found that shRNA knockdown of *STK11* also increased PER1 protein levels (Fig. 1E). Furthermore, in A549 lung cancer cells, which are *STK11*-mutant, addback of wildtype LKB1 was sufficient to reduce PER1 levels (Fig. 1F). These data suggest that LKB1 acts to restrict *PER1* expression and that loss of LKB1 is sufficient to increase *PER1* mRNA and PER1 protein levels.

To further explore the association between LKB1 and PER1 in lung cancer we used publicly available human lung cancer datasets to investigate how *STK11* mutation status related to *PER1* expression in lung adenocarcinoma. Mutations in *STK11* often co-occur with mutations in *KRAS,* and these KRAS/LKB1-mutant (KL) tumors have distinct biology, prognosis, and treatment response than KRAS-mutant tumors with wildtype LKB1 (K). In the Cancer Genome Atlas Lung Adenocarcinoma (TCGA LUAD) dataset (Hoadley et al. 2018), we found that, within *KRAS*-mutant tumors, those with *STK11* mutations had higher *PER1* mRNA expression (p = 0.0224) than those with wildtype LKB1 (Fig. 1G). To explore the effect of *STK11* mutation on PER1 protein levels, we conducted the same K versus KL analysis in samples from the 2020 Clinical Proteomic Tumor Analysis Consortium (CPTAC) study, which includes proteomics data, (Gillette et al. 2020) and again found a significant increase (p = 0.0191) in PER1 protein levels in *KRAS*/*STK11*-mutant tumors (Fig. 1H). We then analyzed these same lung cancer datasets irrespective of driver mutation status, grouping samples solely by *STK11*-WT or *STK11*-mutant status. In the TCGA LUAD dataset, *STK11*-mutant tumors had higher expression (p < 0.0001) of *PER1* mRNA than *STK11*-WT tumors (Fig. 1I). Similarly, in the 2020 CPTAC dataset PER1 protein levels were higher (p = 0.0422) in *STK11*-mutant tumors as compared to *STK11*-WT tumors (Fig. 1J). In agreement with our HBEC3-KT spheroid model, division of the TCGA LUAD dataset into *KRAS*/*STK11*-mutant and *KRAS*/*TP53*-mutant groups also shows lower *PER1* expression in the *TP53*-mutant group (Figure S1B), though this comparison is not statistically significant in the smaller 2020 CPTAC dataset (Figure S1C). Taken together, these data suggest that the higher *PER1* mRNA and PER1 protein expression we observed in cell models lacking LKB1 is also occurring in human lung adenocarcinoma tumors with mutations in *STK11*.

### *PER1* knockdown decreases cell growth and proliferation in LKB1-null cells

**Fig. 2:**
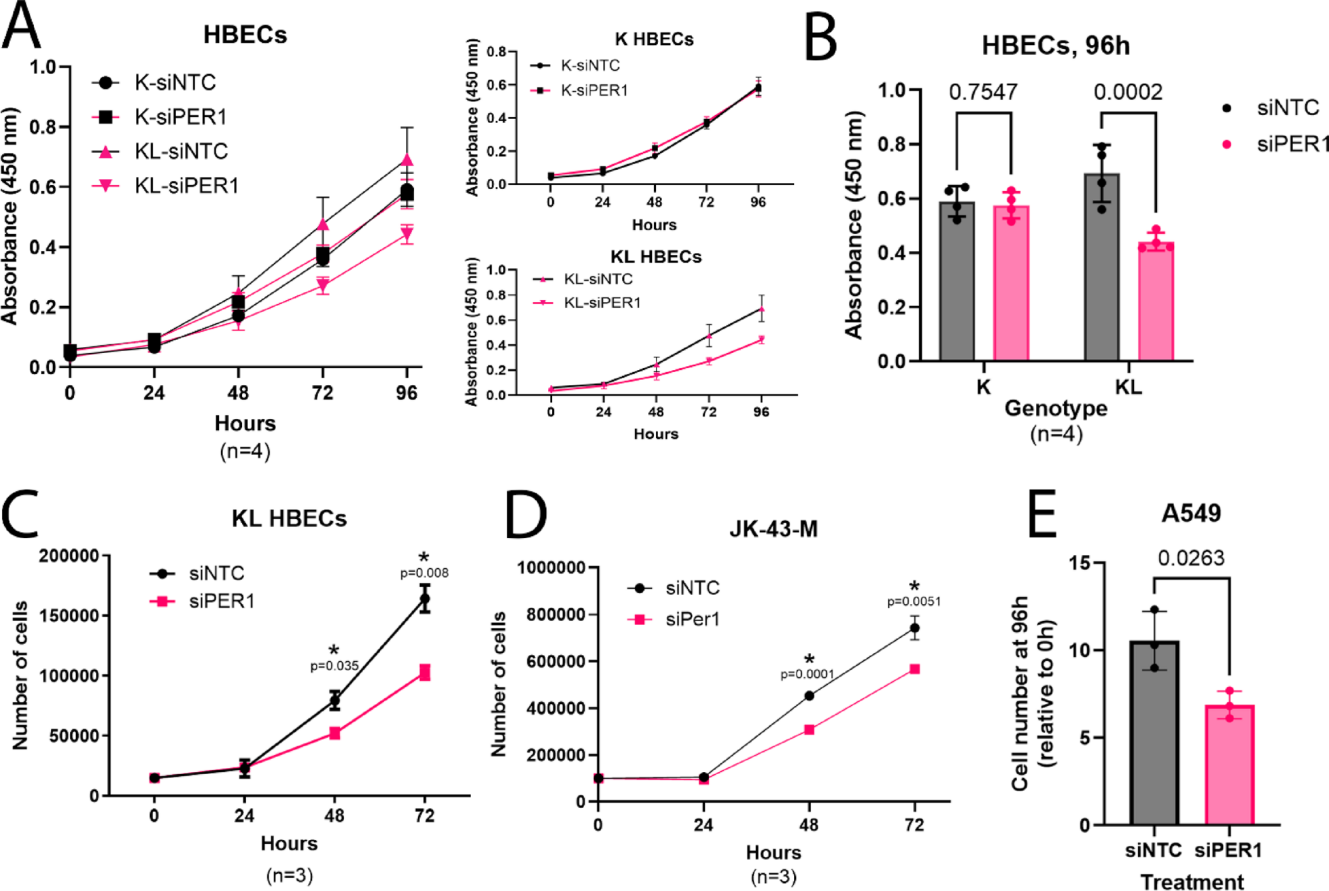
PER1 knockdown decreases proliferation of cells lacking LKB1. **A** Left, CCK-8 cell proliferation assay in *KRAS*^G12V^ (K) and *KRAS*^G12V^/sh*STK11* (KL) HBEC3-KT cells treated with siRNA for non-targeting control (NTC) and *PER1* (si*PER1*). Right, Alternate presentation of the same growth curves, showing K and KL separately. **B** Statistical analysis of growth curves from A, showing the effect of si*PER1* versus siNTC on cell proliferation of K (two-tailed Student’s t-test, p = 0.7547) and KL (two-tailed Student’s t-test, p = 0.0002) HBEC3-KT cells at 96 h. **C** Cell counting cell proliferation assay of siNTC- and si*PER1*-treated KL HBEC3-KT cells; two-tailed Student’s t-test with correction for multiple comparisons, p = 0.035 at 48 h and p = 0.008 at 72 h. **D** Cell counting cell proliferation assay of siNTC- and si*Per1*-treated JK-43-M cells; two-tailed Student’s t-test with correction for multiple comparisons, p = 0.0001 at 48 h and p = 0.0051 at 72 h. **E** Effect of siPER1 versus siNTC on relative cell number of A549 cells at 96 h; two-tailed Student’s t-test, p = 0.0263

LKB1 is a well characterized negative regulator of cell growth and proliferation (Shorning and Clarke 2016); therefore, we investigated the effect of *PER1* knockdown on growth and proliferation in our cell line models. We found that knockdown of *PER1* in our HBEC3-KT models via siRNA had no effect on the growth of *KRAS*^G12V^-expressing cells (p = 0.7547), but did significantly decrease growth of *KRAS*^G12V^/sh*STK11* cells (p = 0.0002), suggesting that *PER1* deregulation may occur early in the transformation process (Fig. 2A–B). We also conducted a direct cell proliferation assay and again found that siRNA knockdown of *PER1* decreased the proliferation of *KRAS*^G12V^/sh*STK11* HBEC3-KT cells at 48 (p = 0.035) and 72 (p = 0.0082) hours (Fig. 2C). To explore a different model, we made use of a cell line, JK43-M, derived from a metastatic lesion from our lab’s genetically-engineered mouse model of *Kras*/*Stk11*-mutant lung adenocarcinoma (Gilbert-Ross 2017;Richardson 2018) and again found that siRNA knockdown of *Per1* decreased cell proliferation at 48 (p = 0.0001) and 72 (p = 0.0051) hours (Fig. 2D). We also sought to determine the impact of *PER1* knockdown in the *STK11*-mutant A549 human lung cancer cell line. Confirming our prior findings, we observed that siRNA knockdown of *PER1* decreased cell proliferation in A549 cells (p = 0.0263; Fig. 2E). These results suggest that the increased *PER1* expression that results from LKB1 loss functionally impacts cell proliferation, both in early oncogenesis and in transformed cells.

### *PER1* knockdown decreases 3D collective invasion in H1299 leader cells

**Fig. 3:**
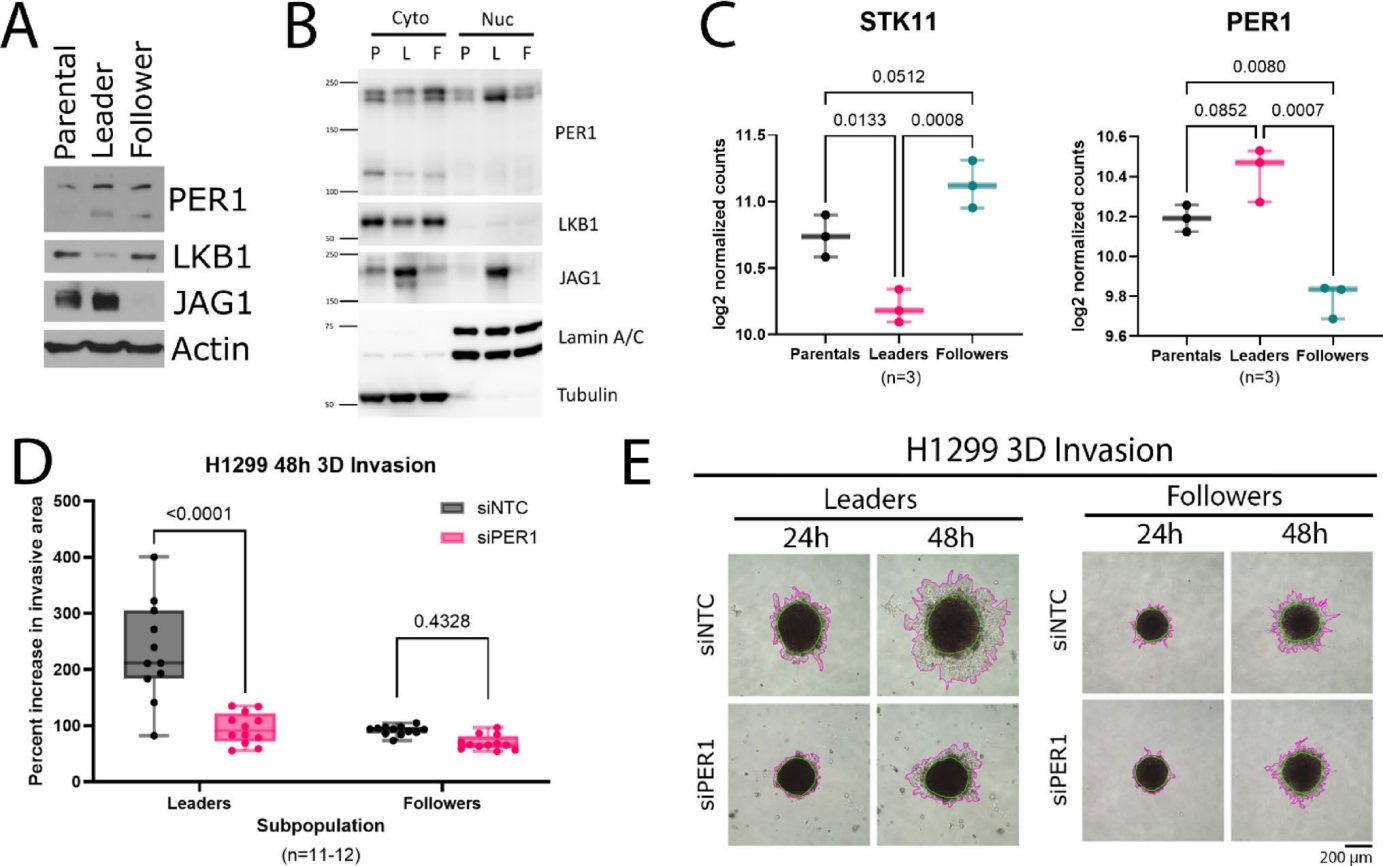
PER1 knockdown decreases invasion in low-LKB1 H1299 leader cells. **A** Western blot of PER1, LKB1, JAG1, and Actin in Parental, Leader, and Follower H1299 cells. **B** Western blot PER1, LKB1, JAG1, Lamin A/C, and Tubulin in cytosolic (cyto) and nuclear (nuc) subcellular fractionation lysates from Parental (P), Leader (L), and Follower (F) H1299 cells. **C** Left, *STK11* mRNA expression in Parental, Leader, and Follower H1299 cells; one-way ANOVA with multiple comparisons tests, Parental vs Leader p = 0.0133, Parental vs Follower p = 0.0512, Leader vs Follower p = 0.0008. Right, *PER1* mRNA expression in Parental, Leader, and Follower H1299 cells; one-way ANOVA with multiple comparisons tests, Parental vs Leader p = 0.0852, Parental vs Follower p = 0.0080, Leader vs Follower p = 0.0007. **D** Effect of si*PER1* versus siNTC on 48 h 3D invasion of Leader and Follower H1299 spheroids (n = 11–12); 2-way ANOVA with multiple comparisons, Leaders p < 0.0001, Followers p = 0.4328. **E** Representative images from H1299 spheroid invasion assays. Green lines indicate the spheroid cores and pink lines indicate the invasive edges

Because the main driver for lung cancer mortality is metastasis, we used invasive ‘leader’ and non-invasive ‘follower’ cells from the parental H1299 cell line to investigate the role of *PER1* in lung cancer collective cell invasion. As opposed to our HBEC3-KT and A549 cell lines, which invade poorly or by mechanisms less relevant to metastasis in 3D assays, H1299 parental, leader, and follower cells are a well-established in vitro model of collective cell invasion with clear relevance to metastasis (Konen et al. 2017; Khatib et al. 2023). Interestingly, it was recently reported that H1299 leader cells are unable to respond to nutrient stress and express low levels of LKB1 protein as compared to the parental and follower populations (Khatib et al. 2025). We first confirmed that leader cells express low levels of LKB1 (Fig. 3A) under optimal nutrient conditions in 2D culture, then used subcellular fractionation to see that, while total PER1 levels are similar in leader and follower subpopulations (Fig. 3A), nuclear localization of PER1 is much higher in leader cells (Fig. 3B). This nuclear PER1 is of particular importance because the main function of PER1 as a repressor of CLOCK/BMAL1-driven gene expression requires its nuclear localization. We then discovered that leader cells have significantly lower expression of *STK11* mRNA as compared to parental (p = 0.0133) and follower (p = 0.0008) subpopulations (Fig. 3C), suggesting that *STK11* may be subject to transcriptional repression in leader cell populations.

We further show that leader cells phenocopy our LKB1 loss-of-function cell models by expressing higher levels of *PER1* transcript when compared to follower cells (p = 0.0007; Fig. 3C). We next investigated how *PER1* knockdown impacted 3D invasion of leader and follower cells. We found that siRNA knockdown of *PER1* significantly decreased 3D collective invasion of leader cells (p < 0.0001) but had no effect on invasion in follower cells (p = 0.4328) (Fig. 3D–E). These results suggest that increased *PER1* expression contributes to the pro-invasive phenotype of aggressive low-LKB1 lung cancer cell subpopulations.

### High PER1 expression in lung adenocarcinoma patients is associated with *STK11*-mutation, low hypoxia scores, low tumor and immune cell admixture, high tumor purity, and low tumor-associated PD-L1 expression

**Fig. 4:**
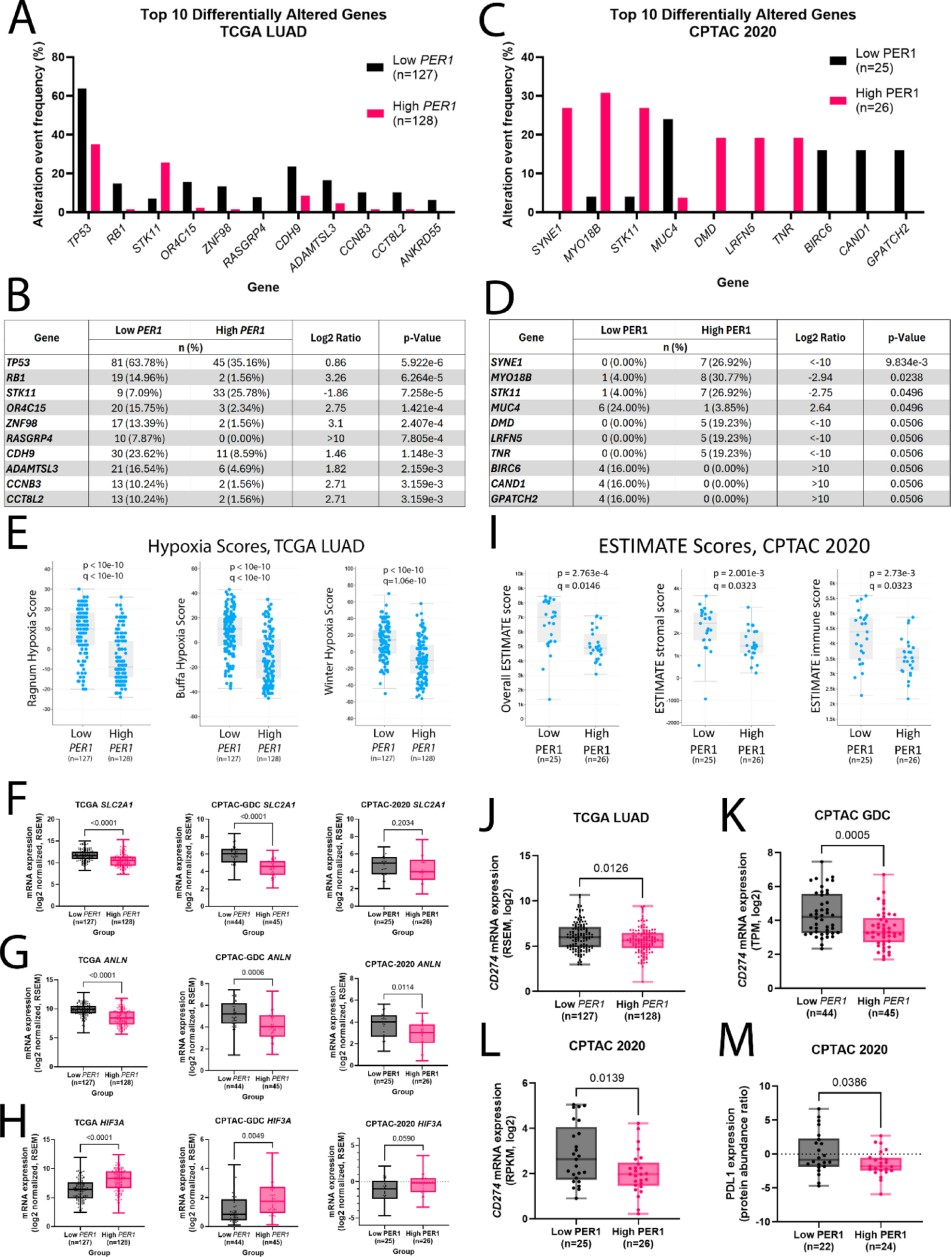
Clinical significance of high PER1 expression in lung adenocarcinoma. **A–B** Top ten most differentially altered genes between the highest and lowest *PER1* mRNA expression quartiles of lung adenocarcinoma samples in the TCGA LUAD study. **C–D** Top ten most differentially altered genes between the highest and lowest PER1 protein expression quartiles of lung adenocarcinoma samples in the CPTAC 2020 study. **E** Ragnum (p < 10^−10^, q < 10^−10^), Buffa (p < 10^−10^, q < 10^−10^), and Winter (p < 10^−10^, q = 1.06^−10^) hypoxia scores as calculated in cBioPortal in Low *PER1* versus High *PER1* mRNA expression quartiles in the TCGA LUAD study. **F** Expression of *SLC2A1* (GLUT1) in: Low *PER1* and High *PER1* expression quartiles in the TCGA LUAD study (two-tailed Student’s t-test, p < 0.0001), Low *PER1* and High *PER1* expression quartiles in the CPTAC-GDC study (two-tailed Student’s t-test, p = 0.0006), and Low PER1 and High PER1 expression quartiles in the CPTAC 2020 study (two-tailed Student’s t-test, p = 0.2034). **G** Expression of *ANLN* in: Low *PER1* and High *PER1* expression quartiles in the TCGA LUAD study (two-tailed Student’s t-test, p < 0.0001), Low *PER1* and High *PER1* expression quartiles in the CPTAC-GDC study (two-tailed Student’s t-test, p = 0.0006), and Low PER1 and High PER1 expression quartiles in the CPTAC 2020 study (two-tailed Student’s t-test, p = 0.0114). **H** Expression of *HIF3A* in: Low *PER1* and High *PER1* expression quartiles in the TCGA LUAD study (two-tailed Student’s t-test, p < 0.0001), Low *PER1* and High *PER1* expression quartiles in the CPTAC-GDC study (two-tailed Student’s t-test, p = 0.0049), and Low PER1 and High PER1 expression quartiles in the CPTAC 2020 study (two-tailed Student’s t-test, p = 0.0590). **I** Overall (p = 2.76^−4^, q = 0.0146), stromal (p = 2.001^−3^, q = 0.0323), and immune (p = 2.73^−3^, q = 0.0323) ESTIMATE scores as calculated in cBioPortal in Low PER1 versus High PER1 protein expression quartiles in the CPTAC 2020 study. **J** *CD274* (PD-L1) mRNA expression in Low *PER1* versus High *PER1* expression quartiles in the TCGA LUAD study; two-tailed Student’s t-test, p = 0.0126. **K** *CD274* (PD-L1) mRNA expression in Low *PER1* versus High *PER1* mRNA expression quartiles in the CPTAC GDC study; two-tailed Student’s t-test, p = 0.0005. **L** *CD274* mRNA expression in Low PER1 versus High PER1 protein expression quartiles in the CPTAC 2020 study; two-tailed Student’s t-test, p = 0.0139. M) PD-L1 protein expression (as measured by protein abundance ratio) in the Low PER1 and High PER1 protein expression quartiles of the CPTAC 2020 study; two-tailed Student’s t-test, p = 0.0386

Having established that knockdown of *PER1* has functional impacts on the proliferation and invasion of lung cancer cells, we next investigated how *PER1* mRNA and PER1 protein expression levels relate to mutation status in the TCGA LUAD and 2020 CPTAC datasets, respectively. For the TCGA LUAD dataset, Low *PER1* (n = 127) and High *PER1* (n = 128) cohorts were established using the lowest and highest quartiles of *PER1* mRNA expression, respectively. For the 2020 CPTAC dataset, Low PER1 (n = 25) and High PER1 (n = 26) cohorts were defined by the lowest and highest quartiles of PER1 protein abundance, respectively. Supporting our previous findings that LKB1 loss results in higher *PER1* expression, we find that lung adenocarcinoma tumors with high expression of *PER1* mRNA (Fig. 4A–B) or PER1 protein (Fig. 4C–D) are enriched for mutations in *STK11.* In both datasets, *STK11* mutations are among the top three most significantly differentially altered genes between the Low and High *PER1*/PER1 groups. In contrast, in the TCGA dataset the Low *PER1* cohort is enriched for *TP53* mutations (Fig. 4A–B), which is consistent with prior published research showing that *PER* genes act as tumor suppressors in *Kras*/*Trp53*-mutant mouse lung cancer models. *TP53* mutations were present at statistically equal proportions in the High PER1 (15 of 25, or 60%) and Low PER1 (13 of 26, or 50%) cohorts in the 2020 CPTAC dataset (p = 0.577) which suggests that LKB1 regulates PER1 at both the transcriptional and post-transcriptional levels. We next investigated the clinical features of the Low and High PER1 cohorts (Supplementary Tables 1–2). In the TCGA LUAD dataset, three independent hypoxia scores were significantly altered between the High *PER1* and Low *PER1* cohorts, with all three hypoxia scores reported in cBioPortal (Ragnum (p < 10e−10, q < 10e−10) (Ragnum et al. 2015), Buffa (p < 10e−10, q < 10e−10) (Buffa et al. 2010), and Winter (p < 10e−10, q = 1.06e−10) (Winter et al. 2007)) being significantly lower in the tumors with high mRNA expression of *PER1* (Fig. 4E). This result was surprising, considering that higher hypoxia scores are generally considered to be negatively prognostic, as are the *STK11* mutations that are enriched in the High *PER1* group. Comparison of the Ragnum, Buffa, and Winter hypoxia score gene sets, and others, showed that *HILPDA*, *P4HA1*, *ADM*, *ALDOA*, *ANLN*, *BNIP3*, *MRGBP*, *CA9*, *CORO1C*, *DDIT4*, *SLC2A1*, *VEGFA*, and *NDRG1* are the most frequent genes appearing in hypoxia gene sets (Giovannantonio et al. 2025). We analyzed expression of this unbiased list of hypoxia-related genes in the Low and High *PER1* expression cohorts in the TCGA LUAD and CPTAC-GDC datasets and the Low and High PER1 expression cohorts in the 2020 CPTAC dataset and found that expression of *SLC2A1,* which encodes GLUT1 and is important for glycolysis, and *ANLN*, which is necessary for cytokinesis and overexpressed in lung adenocarcinoma (Xu et al. 2019), were the most consistently altered hypoxia-related genes in these datasets, with lower expression in the High *PER1/*PER1 groups (Fig. 4F–G). We also investigated whether expression of any hypoxia-inducible factors (HIFs) differed between the *PER1/*PER1 expression cohorts. Consistent with its dependence on post-translational regulation, expression of *HIF1A* did not differ based on *PER1* or PER1 expression in any of the datasets (data not shown). However, expression of *HIF3A*, which is understudied but generally considered to be an inhibitor of hypoxia response (Ravenna et al. 2016), was significantly increased in both the TCGA LUAD and CPTAC-GDC datasets in the High *PER1* groups (p < 0.0001 and p = 0.0049, respectively). These data suggest that high expression of *PER1*, which is known to be involved in hypoxia response along with other clock genes (Chilov et al. 2001; Wu et al. 2017), may impact hypoxia-regulated gene expression through HIF3α.

A different set of clinical attributes, not including hypoxia scores, were available for analysis in the 2020 CPTAC dataset (Supplementary Table 2), and our analysis showed that the clinical Estimation of STromal and Immune cells in MAlignant Tumor tissues using Expression data (ESTIMATE) scores (Yoshihara et al. 2013) were significantly different between the High PER1 and Low PER1 cohorts, with the Stromal (p = 2.001e−3, q = 0.0323) and Immune (p = 2.73e−3, q = 0.0323) ESTIMATE scores significantly lower in tumors with high protein expression of PER1 (Fig. 4I), indicating that immune and stromal cell infiltration into tumor core regions is decreased in PER1 High patients. Accordingly, the Overall ESTIMATE score (p = 2.763e-4, q = 0.0146) was significantly lower in High PER1 patients, indicating a higher level of tumor cell purity. With this result, and considering that *KRAS*/*STK11*-mutant lung cancer displays key differences in tumor associated immune cells as compared to other molecular subtypes and poorer response to immune checkpoint blockade (Skoulidis et al. 2015; Rosellini et al. 2022; Judd et al. 2021; Koyama et al. 2016), we assessed PD-L1 expression (encoded by the gene *CD274*) between the High *PER1* and Low *PER1* and the High PER1 and Low PER1 lung cancer patient cohorts. We found that the High *PER1* and PER1 cohorts had lower expression of the gene encoding PD-L1 compared to the Low *PER1* and Low PER1 cohorts in multiple independent datasets (TCGA LUAD (p = 0.0126), CPTAC GDC (p = 0.0005), and 2020 CPTAC (p = 0.0139)) (Fig. 4J–L). In addition, PD-L1 protein levels were significantly lower in the High PER1 cohort as compared to the Low PER1 cohort in the 2020 CPTAC (p = 0.0356) study, where proteomics data is available (Fig. 4M). Further, analysis of *CD274* expression in our cell line models shows that our LKB1-knockout HBEC3-KT cells, as well as LKB1-low H1299 leader cell subpopulation phenocopies this High PER1-Low *CD274* pattern (Figure S3). We next used MCP-counter, a method of inferring immune cell populations from bulk tumor RNAseq (Becht et al. 2016), to determine whether cohorts expressing high or low levels of *PER1* mRNA or PER1 protein had altered abundance of any immune cell types. In the TCGA LUAD dataset, this analysis revealed an increase in neutrophils (adjusted p = 0.0035) and endothelial cells (adjusted p = 0.0008) (Figure S4). These results, in combination with our results showing enrichment of *STK11* mutation in the High *PER1* cohort, are consistent with previous research showing that STK11 mutation results in increased recruitment of neutrophils to tumors (Koyama et al. 2016). In contrast, different immune cell subtype scores were significantly altered in the 2020 CPTAC dataset, where cohorts were based on PER1 protein expression (Figure S5), including an overall “T cell” score that was decreased in the High PER1 group (adjusted p = 0.0111). Taken together, these data suggest that PER1 may represent an independent predictive biomarker of response to immune checkpoint inhibitors.

## Discussion

### Overview

Loss of LKB1 activity results in de novo resistance to immune checkpoint therapy in lung adenocarcinoma and has widespread impacts on cancer cell biology that necessitate investigation on a subtype-specific basis. The *PER* genes are core components of the circadian clock machinery that have been classified thus far as tumor suppressors, including with respect to their function in the brain’s central circadian clock. Importantly, *PER1* has cancer-relevant roles in cell cycle and DNA damage response which suggests it may have tumor intrinsic roles in modulating the response to anti-cancer therapeutics. In the current study, we use retrospective analysis of human cancer transcriptomic and clinical data in combination with functional studies in human cell models to investigate the significance of altered PER1 expression in LKB1-mutant lung adenocarcinoma.

### Key findings

In this study we show that LKB1 is a negative regulator of *PER1* mRNA and PER1 protein expression, with increased *PER1* mRNA and PER1 protein in immortalized LKB1-deficient lung epithelial cells, and in STK11mutant cancer cells and patient tumors. Knockdown of *PER1* decreases cell growth, proliferation, and invasion in LKB1-deficient models, which suggests that PER1 has an oncogenic role in *STK11*-mutant lung cancer.

In further analysis of human data, we found that, unlike *TP53* mutations which are associated with the Low *PER1* expression group, the High *PER1* and High PER1 groups are associated with *STK11* mutations, significantly lower expression of the gene that encodes PD-L1 (*CD274*), and significantly lower ESTIMATE scores (a measure of immune and stromal cell infiltration and tumor purity).

### Additional findings

Intratumoral hypoxia is generally considered to be poorly prognostic in lung and other cancers (Bhandari et al. 2019; Zhan et al. 2009). In this study we divided lung cancer samples into high and low *PER1* mRNA and PER1 protein expression cohorts and observed lower hypoxia scores in tumors with higher expression of *PER1* mRNA or PER1 protein, the same cohort that is enriched for poorly prognostic *STK11* mutations and low PD-L1 expression. A possible explanation for this is the reciprocal regulation between PER1 and HIF1α (Chilov et al. 2001; Wu et al. 2017; Kobayashi et al. 2017; Hogenesch et al. 1998; Kelly et al. 2020), a potential role for HIF3α, or that hypoxia has context specific roles in molecular subsets of lung adenocarcinoma.

### Limitations

The functional experiments presented here are focused on the tumor intrinsic role of LKB1 on *PER1* mRNA and PER1 protein expression but do not test how loss of LKB1 affects the expression of additional core clock components nor the effect on the oscillation of clock-controlled genes in the lung. Additionally, although we detect a significant association between high *PER1* and PER1 levels and clinical indicators like *CD274* and PD-L1 expression, no experiments were performed to classify high PER1 expression as loss-of-function or gain-of-function, nor to show that the ability of PER1 to modify clinical phenotypes is dependent on its circadian rhythm function. However, our LKB1-WT and LKB1-KO HBEC3-KT cells do show the expected decrease in *CD274* expression with loss of LKB1 and would be a useful model to further explore the functional relationship between LKB1, PER1, and PD-L1.

### Future directions

Future research should focus on two overall questions: what is the mechanism connecting LKB1 and PER1, and can we develop a measure of *PER1* dysregulation for clinical use. In addressing the first question, research should establish the molecular mechanism connecting LKB1 to the regulation of *PER1* expression and establish whether the role of PER1 in LKB1-deficient lung cancer is clock-dependent. LKB1 is chiefly thought of in terms of its master kinase role and among the kinase targets of LKB1 are AMPK and SIK1-3, which are known regulators of circadian clock proteins. This suggests a possible kinase-dependent upstream role for LKB1 in regulation of the circadian clock (Jordan and Lamia 2013; Cho et al. 2019; Um et al. 2011; Lamia et al. 2009; Jagannath et al. 2013, 2023). Interestingly, it is currently unknown which kinases “prime” PERs for further phosphorylation and direction towards either degradation or stabilization and nuclear entry (Hirano et al. 2016). Thus, it is possible that LKB1 directly phosphorylates PER1 to initiate further post-translational modifications, ultimately regulating its protein level and localization. Indeed, there are potential LKB1 target sites within PERs and other clock proteins, as identified in an atlas of human serine/threonine kinase substrates (Johnson et al. 2023). However, LKB1 also has kinase-independent roles in cell motility and transcriptional regulation of the cell cycle (Wilkinson et al. 2017; Konen et al. 2016; Scott et al. 2007) and kinase-dead mutants of LKB1 are recruited to the promoter of Cyclin D1, itself a circadian clock-controlled gene, to promote its transcription. Thus, it is also reasonable to investigate whether LKB1 is directly involved in regulating expression of PER1 or other clock-controlled genes in a kinase-independent manner.

With respect to the second question, while it is not feasible to directly measure the rhythmicity of *PER1* expression in the clinic, it is theoretically possible to establish how gene expression patterns are different between tissues with functional (or rhythmic) and non-function (or arrhythmic) circadian clocks. With this understanding, a multi-gene signature could be used to develop a single-timepoint measure of disruption (or arrhythmicity) in *PER1* expression, which could then be feasibly deployed in the clinic.

## Conclusion

In this study, we report that PER1 is upregulated after loss of LKB1 at the mRNA and protein level, and that this increase in PER1 expression has functional implications for cell proliferation and invasion, as well as an association with biomarkers of poor response to immune checkpoint blockade. This study suggests that PER1 may be a context specific oncogene in LKB1-deficient human lung cancer, underscoring the importance of considering mutational context in studies of lung cancer and chronotherapy.

## Supplementary Information

Below is the link to the electronic supplementary material.

Supplementary file 1 (DOCX 1708 kb).

## Data Availability

The RNA sequencing data analyzed in this study are available as follows: 3-D spheroid HBECs (NCBI Gene Expression Omnibus, GSE271368); H1299 parental, leader, and follower subpopulations (NCBI Sequence Read Archive, PRJNA542374); and synced time course of LKB1-WT and LKB1-KO cells (NCBI Sequence Read Archive, PRJNA1258988).

## Declarations

### Translational relevance

Clinical data support the negative prognostic impact of *STK11* mutations in metastatic non-small cell lung cancer but using STK11 genomic status to guide treatment response has not gained widespread use. We show that knockdown and genetic deletion of LKB1/*STK11* in lung cells are sufficient to upregulate expression levels of *PERIOD 1* (*PER1*) mRNA and protein, and that knockdown of *PER1* in LKB1-mutant cell models decreases tumor growth, proliferation, and invasion. In addition, high levels of *PER1* in lung adenocarcinoma patients are associated with mutations in *STK11*, a decrease in *CD274* (PD-L1), and lower hypoxia and tumor/stromal ESTIMATE scores—whereas low PER1 levels are associated with TP53 mutations in the TCGA cohort. Our results uncover *PER1* as a potential subtype-specific biomarker for response to immunotherapy. Ultimately this could replace the need to use *TP53* and *STK11* genomic status to guide treatment decisions.
