# Supplementary material for: High PER1 expression is associated with STK11 mutation and clinical biomarkers of immunotherapy resistance in lung adenocarcinoma
Source: J Cancer Res Clin Oncol. 2025 Jul 26;151(7):223. doi: 10.1007/s00432-025-06269-9 (PMC12297223; doi:10.1007/s00432-025-06269-9)
Supplement: Supplementary file 1 — (DOCX 1708 kb). [file 432_2025_6269_MOESM1_ESM.docx]

Supplemental Figure 1: *PER1* expression in HBEC3-KT cells and human lung cancer samples taking P53 status into account.


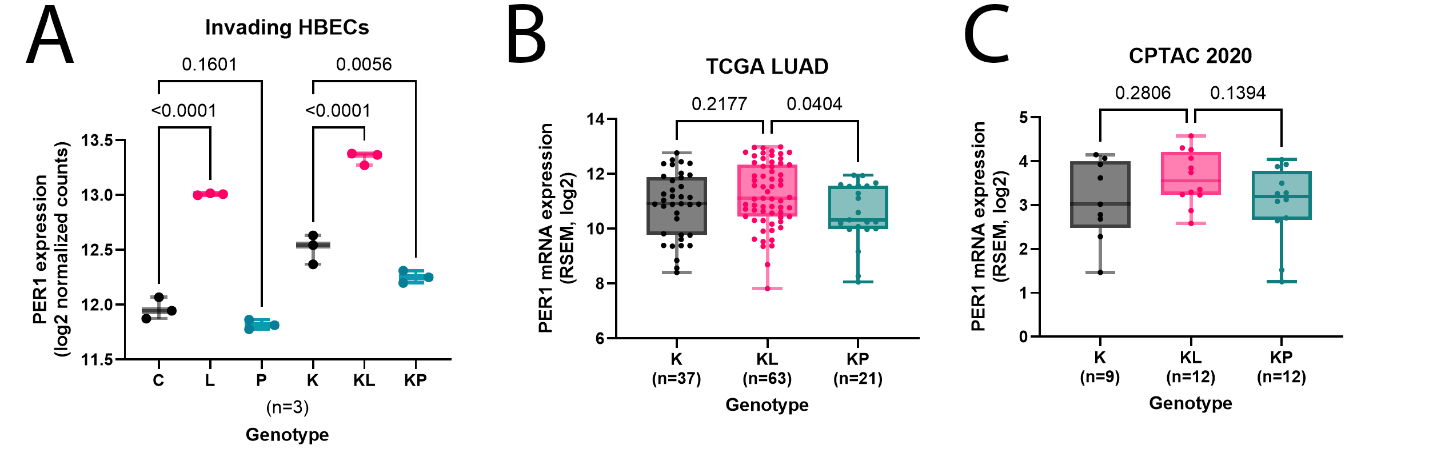


S1 legend:

1. *PER1* mRNA expression in control (C), sh*STK11* (L), KRAS^G12V^ (K), and KRAS^G12V^/sh*STK11* (KL) invading HBEC3-KT organoids (as presented in the main text’s Figure 1) with the addition of sh*TP53* (P) and KRAS^G12V^/sh*TP53* (KP); ordinary one-way ANOVA with Šídák's multiple comparisons tests, C vs L p<0.0001, K vs KL p<0.0001, C vs P p=0.161, K vs KL p=0.056.
2. *PER1* mRNA expression in *KRAS*-mutant (K, n=37), *KRAS*/sh*STK11*-mutant (KL, n=63), and *KRAS*/*TP53*-mutant (KP, n=21) samples in the TCGA LUAD study.
3. *PER1* mRNA expression in *KRAS*-mutant (K, n=37), *KRAS*/sh*STK11*-mutant (KL, n=63), and *KRAS*/*TP53*-mutant (KP, n=21) samples in the 2020 CPTAC study.

Supplemental Figure 2: Validation of anto-PER1 antibody and PER1 molecular weight.


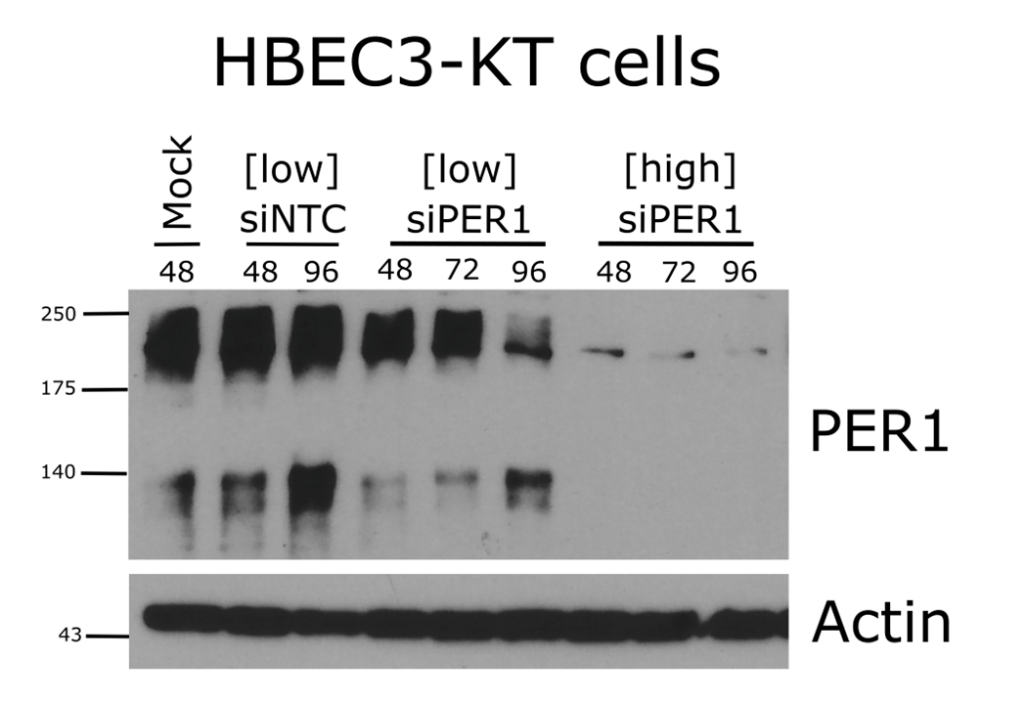


S2 legend:

Western blot of PER1 and Actin in whole-cell lysates of HBEC3-KT cells with mock treatment (no siRNA) or treated with a low or high concentration of non-targeting control (siNTC) siRNA or siRNA targeting *PER1,* and collected at 48, 72, or 96 hours post-siRNA treatment, as indicated. Note that si*PER1* treatment depletes both low and high molecular weight bands of PER1.

Supplemental Figure 3: *CD274* (PD-L1) expression in cell line models.


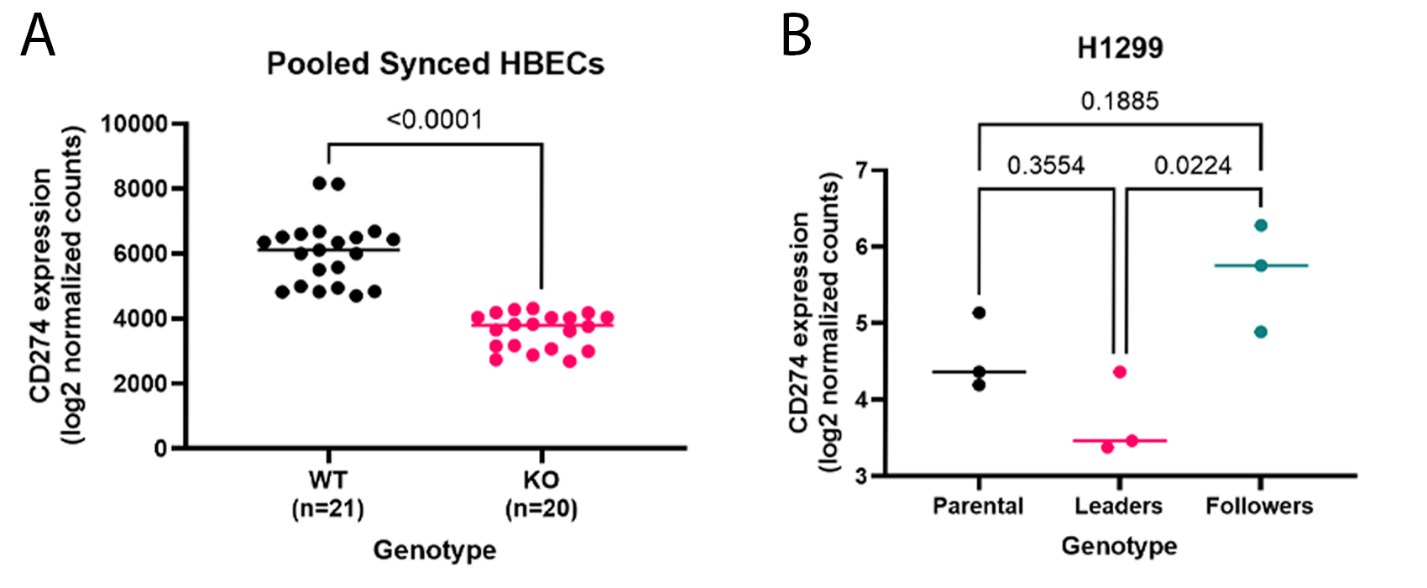


S3 legend:

1. *CD274* mRNA expression in LKB1-wildtype (WT) and LKB1-knockout (KO) synced HBEC3-KT cells, with all timepoints pooled. Unpaired t-test showed lower expression of *CD274* in LKB1-knockout cells (p<0.0001).
2. *CD274* mRNA expression from RNAseq of parental, leader, and follower H1299 cell subpopulations. Adjusted p values from an ordinary one-way ANOVA followed by Šídák's multiple comparisons tests are noted on the figure, with expression of CD274 lower in leaders than in followers (p=0.0224).

Supplemental Figure 4: MCP-counter immune cell type results for TCGA dataset


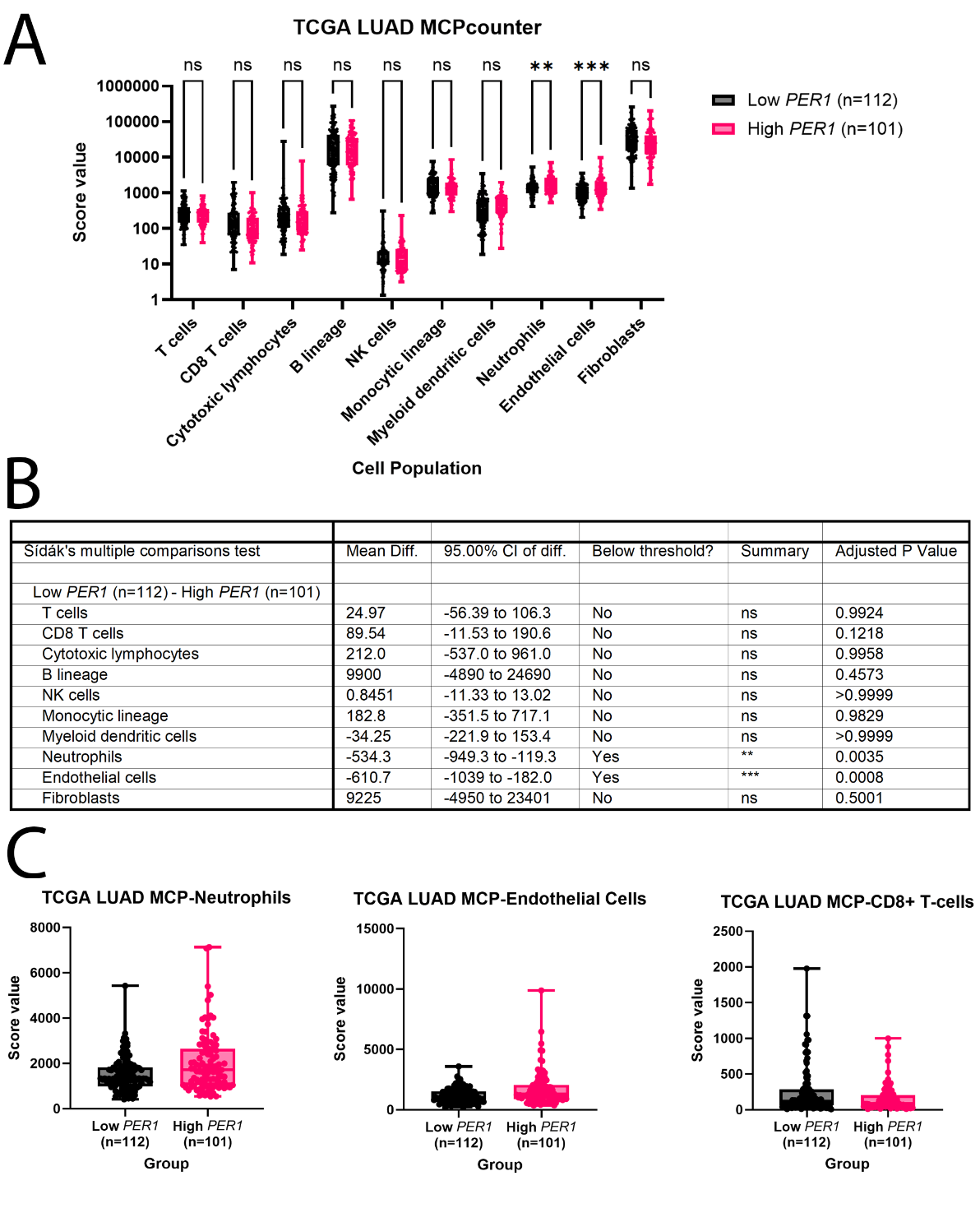


S4 legend:

A. Results in Low *PER1* (n=112) and High *PER1* (n=101) groups for all MCP-counter-scored immune cell types from the TCGA LUAD study’s bulk tumor RNAseq. Two-way ANOVA followed by Šídák's multiple comparisons test found that neutrophil and endothelial cell type scores were significantly different between Low *PER1* and High *PER1* groups. (Note that the scale is logarithmic to allow visualization of lower scores that were overly compacted with a linear scale.)

B. Full table of Šídák's multiple comparisons test results for each cell type score.

C. Graphs of significantly altered (neutrophil and endothelial cell) cell type scores and a nearer to significance cell type (CD8+ T-cells).

Supplemental Figure 5: MCP-counter immune cell type results for CPTAC-2020 dataset


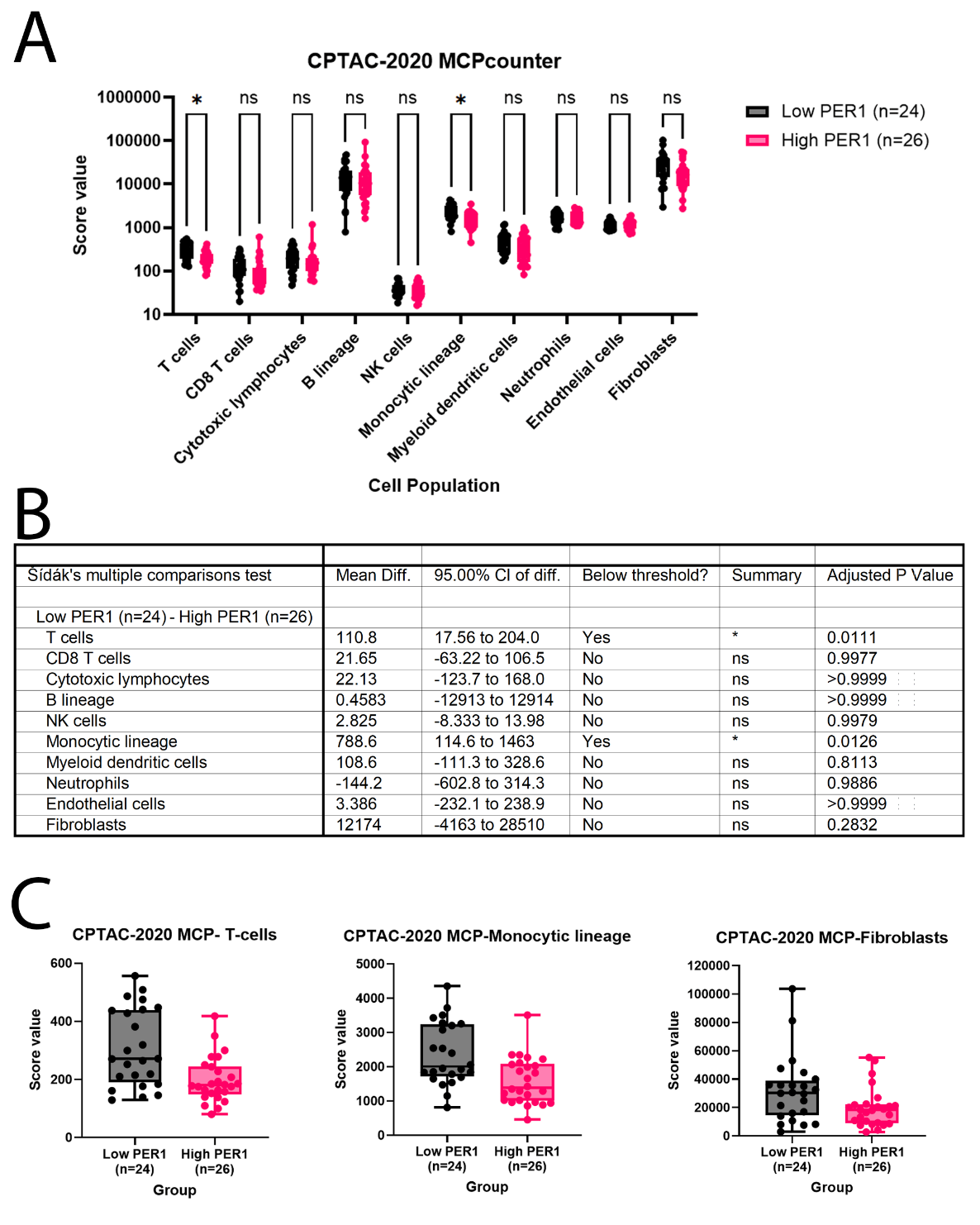


S5 legend:

A. Results in Low PER1 (n=24) and High PER1 (n=26) groups for all MCP-counter-scored immune cell types from the 2020 CPTAC study’s bulk tumor RNAseq. Two-way ANOVA followed by Šídák's multiple comparisons test found that T-cell and monocytic lineage cell type scores were significantly different between Low PER1 and High PER1 groups. (Note that the scale is logarithmic to allow visualization of lower scores that were overly compacted with a linear scale.)

B. Full table of Šídák's multiple comparisons test results for each cell type score.

C. Graphs of significantly altered (T-cell and monocytic lineage) cell type scores and a borderline-significant cell type (Fibroblasts).

Supplementary Table 1, Results of all available clinical attributes in cBioPortal for the TCGA LUAD study, Low *PER1* (n=127) versus High *PER1* (n=128).

| **Clinical Attribute** | **Attribute Type** | **Statistical Test** | **p-Value** | **q-Value** |
| --- | --- | --- | --- | --- |
| Ragnum Hypoxia Score | Patient | Wilcoxon Test | 1.55e-15 | 7.15e-14 |
| Buffa Hypoxia Score | Patient | Wilcoxon Test | 3.34e-13 | 7.67e-12 |
| Winter Hypoxia Score | Patient | Wilcoxon Test | 6.90e-12 | 1.06e-10 |
| Tissue Source Site Code | Sample | Chi-squared Test | 1.732e-4 | 1.593e-3 |
| Tissue Source Site | Sample | Chi-squared Test | 1.732e-4 | 1.593e-3 |
| Tumor Break Load | Sample | Wilcoxon Test | 6.642e-4 | 5.092e-3 |
| Aneuploidy Score | Sample | Wilcoxon Test | 0.0113 | 0.0743 |
| MSIsensor Score | Sample | Wilcoxon Test | 0.0237 | 0.136 |
| Tissue Prospective Collection Indicator | Sample | Chi-squared Test | 0.0646 | 0.297 |
| Tissue Retrospective Collection Indicator | Sample | Chi-squared Test | 0.0646 | 0.297 |
| American Joint Committee on Cancer Tumor Stage Code | Patient | Chi-squared Test | 0.0806 | 0.312 |
| Mutation Count | Sample | Wilcoxon Test | 0.0815 | 0.312 |
| International Classification of Diseases for Oncology, Third Edition ICD-O-3 Site Code | Patient | Chi-squared Test | 0.116 | 0.387 |
| TMB (nonsynonymous) | Sample | Wilcoxon Test | 0.118 | 0.387 |
| ICD-10 Classification | Patient | Chi-squared Test | 0.137 | 0.404 |
| International Classification of Diseases for Oncology, Third Edition ICD-O-3 Histology Code | Patient | Chi-squared Test | 0.141 | 0.404 |
| Form completion date | Patient | Chi-squared Test | 0.172 | 0.464 |
| Last Communication Contact from Initial Pathologic Diagnosis Date | Patient | Wilcoxon Test | 0.256 | 0.607 |
| American Joint Committee on Cancer Publication Version Type | Patient | Chi-squared Test | 0.293 | 0.629 |
| Race Category | Patient | Chi-squared Test | 0.314 | 0.629 |
| Prior Diagnosis | Patient | Chi-squared Test | 0.315 | 0.629 |
| Tumor Type | Sample | Chi-squared Test | 0.367 | 0.682 |
| Fraction Genome Altered | Sample | Wilcoxon Test | 0.413 | 0.682 |
| Birth from Initial Pathologic Diagnosis Date | Patient | Wilcoxon Test | 0.431 | 0.682 |
| Ethnicity Category | Patient | Chi-squared Test | 0.437 | 0.682 |
| Neoplasm Disease Lymph Node Stage American Joint Committee on Cancer Code | Patient | Chi-squared Test | 0.457 | 0.682 |
| Other Patient ID | Patient | Chi-squared Test | 0.471 | 0.682 |
| MSI MANTIS Score | Sample | Wilcoxon Test | 0.489 | 0.682 |
| Diagnosis Age | Patient | Wilcoxon Test | 0.51 | 0.691 |
| Neoplasm Disease Stage American Joint Committee on Cancer Code | Patient | Chi-squared Test | 0.569 | 0.727 |
| Genetic Ancestry Label | Patient | Chi-squared Test | 0.586 | 0.728 |
| In PanCan Pathway Analysis | Patient | Chi-squared Test | 0.609 | 0.737 |
| Radiation Therapy | Patient | Chi-squared Test | 0.626 | 0.739 |
| Sex | Patient | Chi-squared Test | 0.757 | 0.87 |
| Person Neoplasm Cancer Status | Patient | Chi-squared Test | 0.791 | 0.887 |
| New Neoplasm Event Post Initial Therapy Indicator | Patient | Chi-squared Test | 0.909 | 0.963 |
| American Joint Committee on Cancer Metastasis Stage Code | Patient | Chi-squared Test | 0.935 | 0.963 |
| Neoadjuvant Therapy Type Administered Prior To Resection Text | Patient | Chi-squared Test | 0.997 | 0.997 |

Supplementary Table 2, Results of all available clinical attributes in cBioPortal for the CPTAC 2020 study, Low PER1 (n=25) versus High PER1 (n=26).

| **Clinical Attribute** | **Attribute Type** | **Statistical Test** | **p-Value** | **q-Value** |
| --- | --- | --- | --- | --- |
| Overall ESTIMATE score | Sample | Wilcoxon Test | 2.763e-4 | 0.0146 |
| Tumor Purity | Sample | Wilcoxon Test | 4.962e-4 | 0.0146 |
| ESTIMATE stromal score | Sample | Wilcoxon Test | 2.001e-3 | 0.0323 |
| ESTIMATE immune score | Sample | Wilcoxon Test | 2.736e-3 | 0.0323 |
| TSNet Purity | Sample | Wilcoxon Test | 2.736e-3 | 0.0323 |
| NMF Consensus | Sample | Chi-squared Test | 0.0109 | 0.108 |
| STK11 Mutation Status | Sample | Wilcoxon Test | 0.0259 | 0.218 |
| BRAF Mutation Status | Sample | Wilcoxon Test | 0.0431 | 0.318 |
| Smoking Signature Fraction WGS | Patient | Wilcoxon Test | 0.0667 | 0.398 |
| Dnp Gg->Tt Or Cc->Aa Count | Sample | Wilcoxon Test | 0.072 | 0.398 |
| mRNA Expression Subtype TCGA | Sample | Chi-squared Test | 0.0742 | 0.398 |
| EGFR Mutation Status | Sample | Wilcoxon Test | 0.117 | 0.574 |
| Putative Driver Mutation | Sample | Chi-squared Test | 0.134 | 0.606 |
| RB1 Mutation Status | Sample | Wilcoxon Test | 0.149 | 0.626 |
| Mutation Count (DNP) | Sample | Wilcoxon Test | 0.162 | 0.634 |
| RB1 Mutation | Sample | Chi-squared Test | 0.172 | 0.634 |
| Smoking Score WGS | Patient | Wilcoxon Test | 0.197 | 0.684 |
| Stage | Patient | Chi-squared Test | 0.212 | 0.696 |
| Total Mutation Count | Sample | Wilcoxon Test | 0.282 | 0.826 |
| Mutation Count (Excluding INDELs) | Sample | Wilcoxon Test | 0.286 | 0.826 |
| ROS1 Fusion | Sample | Wilcoxon Test | 0.308 | 0.826 |
| EGFR Mutation | Sample | Chi-squared Test | 0.317 | 0.826 |
| ARAF Mutation Status | Sample | Wilcoxon Test | 0.327 | 0.826 |
| Dominant Histological Subtype | Sample | Chi-squared Test | 0.346 | 0.826 |
| KEAP1 Mutation | Sample | Chi-squared Test | 0.368 | 0.826 |
| Mutation Signature Activity W3 COSMIC2 | Sample | Chi-squared Test | 0.394 | 0.826 |
| TP53 Mutation | Sample | Chi-squared Test | 0.396 | 0.826 |
| ALK Fusion | Sample | Wilcoxon Test | 0.418 | 0.826 |
| Mutation Signature Activity W1 COSMIC5 | Sample | Chi-squared Test | 0.433 | 0.826 |
| Mutation Signature Activity W2 COSMIC4 | Sample | Chi-squared Test | 0.433 | 0.826 |
| Aliquot | Sample | Chi-squared Test | 0.434 | 0.826 |
| TP53 Mutation Status | Sample | Wilcoxon Test | 0.477 | 0.88 |
| Dominant Signature WGS notSmoking 50perc | Patient | Chi-squared Test | 0.514 | 0.911 |
| Weight | Patient | Wilcoxon Test | 0.627 | 0.911 |
| Somatic Status | Sample | Chi-squared Test | 0.631 | 0.911 |
| Ethnicity | Patient | Chi-squared Test | 0.631 | 0.911 |
| Height | Patient | Wilcoxon Test | 0.64 | 0.911 |
| Pack Years Smoked | Patient | Wilcoxon Test | 0.644 | 0.911 |
| Region Of Origin | Patient | Chi-squared Test | 0.651 | 0.911 |
| Mutation Count (Somatic) | Sample | Wilcoxon Test | 0.655 | 0.911 |
| mRNA Stemness Index | Sample | Wilcoxon Test | 0.665 | 0.911 |
| Dominant Signature Fraction WGS notSmoking | Patient | Wilcoxon Test | 0.666 | 0.911 |
| Mutation Count | Sample | Wilcoxon Test | 0.677 | 0.911 |
| TMT Channel | Sample | Chi-squared Test | 0.69 | 0.911 |
| Age | Patient | Wilcoxon Test | 0.713 | 0.911 |
| Cigarettes Per Day | Patient | Wilcoxon Test | 0.724 | 0.911 |
| Country Of Origin | Patient | Chi-squared Test | 0.726 | 0.911 |
| BMI | Patient | Wilcoxon Test | 0.742 | 0.911 |
| STK11 Mutation | Sample | Chi-squared Test | 0.767 | 0.911 |
| Smoking History | Patient | Chi-squared Test | 0.772 | 0.911 |
| Experiment | Sample | Wilcoxon Test | 0.836 | 0.945 |
| NMF Cluster Membership | Sample | Wilcoxon Test | 0.858 | 0.945 |
| Sex | Patient | Chi-squared Test | 0.862 | 0.945 |
| TMB (nonsynonymous) | Sample | Wilcoxon Test | 0.865 | 0.945 |
| Smoking Status | Patient | Chi-squared Test | 0.886 | 0.951 |
| CIMP Status | Sample | Chi-squared Test | 0.917 | 0.965 |
| KRAS Mutation Status | Sample | Wilcoxon Test | 0.932 | 0.965 |
| KEAP1 Mutation Status | Sample | Wilcoxon Test | 0.96 | 0.976 |
| Secondhand Smoke | Patient | Chi-squared Test | 0.992 | 0.992 |
